# Supplementary material for: The experiences of UK-Chinese individuals during the COVID-19 pandemic: A qualitative interview study
Source: PLoS One. 2023 Jan 17;18(1):e0280341. doi: 10.1371/journal.pone.0280341 (PMC9844865; doi:10.1371/journal.pone.0280341)
Supplement: S1 File — (DOCX) [file pone.0280341.s001.docx]

**Interview topic guide**

**Opener**

Coronavirus/COVID/COVID 19 interchangeable

How has COVID 19 affected you?

**Experiences**

Any positive experiences during COVID?

Any negative experiences during COVID?

- Isolation
- Other people’s responses
- Changes to your behaviour?

How has lockdown affected you?

**Family and overseas**

Do you have any family or friends overseas?

How do you communicate with your family/friends overseas?

Differences in experiences?

Differences in what you are allowed/not allowed to do?

**The future**

How do you think COVID will affect the UK Chinese in the short term?

How do you think COVID will affect the UK Chinese in the long term?
