# Supplementary material for: The experiences of UK-Chinese individuals during the COVID-19 pandemic: A qualitative interview study
Source: PLoS One. 2023 Jan 17;18(1):e0280341. doi: 10.1371/journal.pone.0280341 (PMC9844865; doi:10.1371/journal.pone.0280341)
Supplement: S1 Table — (DOCX) [file pone.0280341.s003.docx]

**Table of illustrative quotes**

| **Theme** | **Quotes** |
| --- | --- |
| **Attribution of stigma** |  |
| Stigmatisation through (mis)identity | “So once I was on the (train) and I got on and someone started… someone who was sat actually probably about two metres away from me started getting their anti-bacteria gel and kind of like gelling their hands as soon as I got on the (train) and I can’t really say that’s because of me, I don’t know but I probably think the fact that I was Chinese probably had something to do with it.” (Participant 5)  “P At the very beginning of the pandemic I had to go to the surgery, the clinic, ‘cos I’ve been worried that’s why I have to put the face mask on. As I walk into the clinic, two adults they come at me, calling me name as coronavirus.  I Oh no, I’m so sorry to hear that. Do you think that was how you looked or do you think it might, you know, it may be the mask or-?  P Yeah I think both. Think the Chinese having the mask.” (Participant 9)  “my colleague, she’s Chinese, because she’s got some spare masks, face masks, she thought she… first of all she would like to go and donate to the hospital or everywhere for the people to use but obviously that’s fine but she also… when she share or ask one of her colleagues, her colleague said… because she said oh I’ve got some face masks I would like to give this hospital or give this department if we need it, and the one she asked, and ask her is like oh is that clean or you used? Oh my colleague was… she was so, so upset.” (Participant 3)  “A lot of Chinese students in university they got verbally abused and even some students they got beaten up in the residential hall, about a month ago probably and they report it to us and we helped them to report to the police.” (Participant 14)  “I’m fully aware of the anti-social or hate incidents because we heard of it within the Chinese Association. As a result we try to keep a distance with other people because you don’t have a close contact with them you won’t get a problem. Also we only go out in the day time. We’re not putting ourselves in jeopardy at night.” (Participant 15)  “they get spat on, they get verbal abuse, some of them get stones thrown because (inaudible 1:02:02) in those hate crime never… I know, I have the report, I report to the police on their behalf and luckily the lockdown reduced the number of cases because nobody go out.” (Participant 8)  “I Did you find that other people were ok with you wearing the mask or-  P I think one or two people did look at me.  I Really?  P I don’t know whether they tried to move away, I don’t know, but one or two people did look at me.  I And why do you think that that was? Why did they behave like that?  P Don’t know, they just found it…Well I don’t know, they… I don’t know. I’m not worried at all. Protecting myself or protecting somebody else.” (Participant 12) |
| Markers of pandemic awareness | “I know since January, because some of Chinese kids, students, they have more knowledge or more concern about this because what happened in China in Wuhan and they start wearing masks in cities and they get spat on, they get verbal abuse, some of them get stones thrown” (Participant 8)  “At the very beginning of the pandemic I had to go to the surgery, the clinic, ‘cos I’ve been worried that’s why I have to put the face mask on. As I walk into the clinic, two adults they come at me, calling me name as coronavirus.” (Participant 9)  “No, because, you know, that’s happening in China before England so most of Chinese once they happen the family send some masks from China because obviously… they couldn’t, and they sold out in the UK, so they couldn’t get anything so they… or most families send or all most friends send, so every Chinese family I think they have spare.” (Participant 3)  “P Yes I have. I’ve family and friends in Hong Kong.  I And how are things for them?  P Well they seem to be ok. They’re still going out, they go shopping every day, they still eat out but I think the main difference is they wear masks everywhere whereas in the West, or certainly in the UK, I don’t see many of them wearing masks which I don’t understand, which totally is baffling as to why it isn’t something compulsory to be doing that whereas its compulsory in China and Hong Kong” (Participant 6)  “P I think they should wear mask. Normally they might not. I mean recently they open the restaurants or preparing takeaway and things like that, they’re on television and the people who prepare the food did not wear mask.  I Why do you think they weren’t wearing masks?  P I don’t know. They should but I don’t know why they’re not.” (Participant 12)  “actually first they will be worried about me a lot because they think all the people will go out without wearing face mask, they think less behaviour will be very dangerous and my parents even talk us maybe if possible and all of you can come back to China” (Participant 2)  “if we’re kind of wearing masks on the street whilst walking, not for exercise, and most people aren’t wearing masks whereas they see you wearing a mask they might kind of feel suspicious as if you have been infected or as if you have a problem. They kind of seem less comfortable ‘cos nobody else is wearing it.” (Participant 1) |
| **Pandemic legacies** |  |
| Legacies of previous pandemics | “P I do this without the government guidance because of my age and I know face masks will help me that’s why I put it on.  I How did she know that a face mask would help her?  P This information’s come from various areas, from the TV, from the magazine for the Chinese Association, we receive this information from quite a few different areas.  (Participant 11)  “In my mind if I have the disease I’ll stop spreading it to other people, if I don’t have the disease I stop catching it and if everybody wearing it I think the chance to get is very, very minimal.” (Participant 8)  “I think they’ve… I think having had previous experience with SARS and MERS as a country I think they don’t want a repeat of what’s happened so I think people were probably a bit more compliant with lockdown and they were quite clear in the message that they were sending out to people” (Participant 5)  “P Well even before the coronavirus, even if you had a cold or something and you were travelling on public transport, people would just… its natural, it’s something natural that they would do, just put on their mask. Not just to protect them, it’s to protect the others, especially when you sneeze and cough  …..  I Ok. And has that been around for a while or-?  P It’s been around since SARS I think. 2003, yeah. (Participant 6)  “I think when people reflect on this whole pandemic I think it might be an idea to see what the other Asian countries are doing ‘cos yes most of these Asian countries would have the benefit of experience from SARS but they seem to have mobilised and managed to keep the figures down.” (Participant 7)  “Because of the SARS in 2003, yeah the first experience of happening in Hong Kong in 2003. So people learn about it. Quite a lot of people died during that year so when coronavirus came people started to get alert” (Participant 14)  “I think it’s just because everyone in Hong Kong that they’ve spoken to, a lot of the news, a lot of the people on the news that you see, people wearing masks so I think they thought that that might be something that could protect them. So they were quite keen to get masks quite early on.” (Participant 5)  “I So they were wearing face masks even before Covid or only-  P No, no, no. Before the Covid you probably remember or not aware in 2003 there was another virus called SARS. They suffer big time. Since then people happy to wear face mask…” (Participant 8) |
| Ascription of blame | “P it’ll take a long time before they go back to normal because one of the things is it comes from the Chinese (inaudible 51:52).  I So they-  P Even though when they said that it came from a market, they can’t actually prove its from there. Officially that’s where they said it was, you see these animal, but I don’t think they ever prove it and because it’s reported in the beginning and it is stuck, that Chinese did everything and from there. You can’t wash it off.  I You feel like-  P So therefore when they start opening all the catering, they would be stuck with it and people’s confidence, the first they say Chinese meal, oh yes any kind of animal, isn’t it? It’ll take a while, I’m sure, but it’s not a good time to be in business and not just Chinese I suppose, it’s all… everything when it comes back, it’ll take a long time to get back.” (Participant 4)  “it was first reported in China but I think it’s dangerous to say that it’s a Chinese virus, ok, and I’m just referring to some high political leader who might have said that, and I’m sure that a lot of people have… but it’s dangerous when you put an ethnicity to an illness because first of all you’re… when you’re doing that you’re just basically putting like a community under not a very nice spotlight. Not a very nice spotlight.” (Participant 7)  “P Now they’re… it’s a thing now isn’t it, they call it Chinese virus.  I And is that something you felt, that kind of stigma?  P A little bit, yes. It doesn’t help when you’re here, see the BBC reporting all the time when they introduce it saying that the virus where it started off from China.” (Participant 4)  “I was reading all about these backlashes against Asian Americans, it made for very uncomfortable reading. Very, very uncomfortable reading.” (Participant 7)  “I kind of worried about what would happen, you know, if patients saw me in hospital, how comfortable they would feel if they knew that I was treating them, if maybe they would say oh I don’t want someone who is Chinese to treat me because a lot of… because coronavirus kind of started in China and then people… and there was a lot of media around Trump saying it was the Chinese virus, I feel like that hasn’t really been great for the Chinese community, so I do worry about what would patients think, would they be comfortable if I was involved in their care, would they think that I was trying to spread the virus, do they think that I had like, you know, those kind of things which are obviously untrue but I think because when people get scared they do sometimes have irrational thoughts or things are reported in a certain way, I think it can have negative effects.” (Participant 5) |
| **Individual versus societal responses** |  |
| Extent of freedom | “They are quite different because I suppose the Chinese government has more power and authority over the decisions whereas here it’s more democratic so I suppose the decision making is more difficult to decide and enforce the restrictions on every day kind of movement and also to make decisions to lockdown.” (Participant 1)  “I think the response was a bit more vague and a bit more kind of like it’s up to you to do your bit rather than you need to stay indoors, you can’t go out, kind of, yeah, leadership really.” (Participant 5)  “Mostly the best (sic British) people they have a lot of freedom so they don’t follow the government” (Participant 3)  “The government has imposed laws, anyone is spitting they are fined. If they don’t wear mask they are fined…they would be able to go out only for one hour in total to go shopping and come back so it was a political order to people from eight to three would go a certain date, certain time, so if someone is out and the police ask them to show the ticket of the time” (Participant 13)  “the funeral was at the end of month and he [uncle R] had to go back to Hong Kong for a 14 days quarantine… and he’s been tagged as well. He can’t leave the flat for 14 days.…They know where he is” (Participant 4) |
| Implicit faith in government | “I think maybe because of the culture and the (inaudible 45:56) of the country is different. So it’s hard to say which one is right but I think the government will make the decisions, will think very careful about all the people’s profit or benefits, so I will follow the government” (Participant 1)  “we have to just accept or respect and also follow their command or decision and do whatever they tell us to do.” (Participant 3)  “If the government comes out with guidelines wear a face mask then yes, people would accept it because… During the time of crisis, everyone’s listening to the government, everyone wants to do their part and everyone is… we’re all trusting our governments to help us through this crisis and if they came out with the guidelines about face masks then without a question everyone will accept it.” (Participant 7) |
