# Supplementary material for: The experiences of UK-Chinese individuals during the COVID-19 pandemic: A qualitative interview study
Source: PLoS One. 2023 Jan 17;18(1):e0280341. doi: 10.1371/journal.pone.0280341 (PMC9844865; doi:10.1371/journal.pone.0280341)
Supplement: Attachment — Submitted filename: Response to reviewers.docx [file pone.0280341.s004.docx]

**We are grateful for the opportunity to improve our manuscript. We have provided responses to the academic editors’ and reviewers’ comments below.**

ACADEMIC EDITORS COMMENTS

When submitting your revision, we need you to address these additional requirements.

1. Please ensure that your manuscript meets PLOS ONE's style requirements, including those for file naming. The PLOS ONE style templates can be found at

<https://journals.plos.org/plosone/s/file?id=wjVg/PLOSOne_formatting_sample_main_body.pdf> and

<https://journals.plos.org/plosone/s/file?id=ba62/PLOSOne_formatting_sample_title_authors_affiliations.pdf>

**Authors’ response: Thank you. We have checked our manuscript to ensure it meets PLOS ONE’s style requirements.**

2. Please include your full ethics statement in the ‘Methods’ section of your manuscript file. In your statement, please include the full name of the IRB or ethics committee who approved or waived your study, as well as whether or not you obtained informed written or verbal consent. If consent was waived for your study, please include this information in your statement as well.

**Authors’ response: Thank you. We have updated the methods section regarding ethical approval so it now reads *“Ethical approval was granted from the University of Bristol Faculty of Health Sciences Research Ethics Committee (reference number 94685).”* We have also amended the methods section regarding provision of consent and this now reads: *“All participants provided informed verbal consent to participation which was audio-recorded and transcribed.”.***

3. We note that the grant information you provided in the ‘Funding Information’ and ‘Financial Disclosure’ sections do not match.

When you resubmit, please ensure that you provide the correct grant numbers for the awards you received for your study in the ‘Funding Information’ section.

**Authors’ response: Thank you for pointing out this error. We have updated the funding information section to match the financial disclosure, and also corrected the ordering. To clarify the funding, Katie Wong received a grant from the Elizabeth Blackwell Institute, University of Bristol to conduct this research. This grant was derived from a Wellcome Trust block grant to the Elizabeth Blackwell Institute.**

4. In your Data Availability statement, you have not specified where the minimal data set underlying the results described in your manuscript can be found. PLOS defines a study's minimal data set as the underlying data used to reach the conclusions drawn in the manuscript and any additional data required to replicate the reported study findings in their entirety. All PLOS journals require that the minimal data set be made fully available. For more information about our data policy, please see <http://journals.plos.org/plosone/s/data-availability>.

Upon re-submitting your revised manuscript, please upload your study’s minimal underlying data set as either Supporting Information files or to a stable, public repository and include the relevant URLs, DOIs, or accession numbers within your revised cover letter. For a list of acceptable repositories, please see <http://journals.plos.org/plosone/s/data-availability#loc-recommended-repositories>. Any potentially identifying patient information must be fully anonymized.

Important: If there are ethical or legal restrictions to sharing your data publicly, please explain these restrictions in detail. Please see our guidelines for more information on what we consider unacceptable restrictions to publicly sharing data: <http://journals.plos.org/plosone/s/data-availability#loc-unacceptable-data-access-restrictions>. Note that it is not acceptable for the authors to be the sole named individuals responsible for ensuring data access.

We will update your Data Availability statement to reflect the information you provide in your cover letter.

**Authors’ response: Thank you. We have now uploaded anonymised interview transcripts to the University of Bristol’s Research Data Repository. Access to these data is controlled, given the personal and sensitive nature of topics discussed and the fact that we cannot rule out the risk of identification, even after anonymisation. Individual researchers will need to gain approval to access to the controlled data through the University of Bristol via the Data Access Committee (DAC), before data can be shared after their host institution has signed a Data Access Agreement. We have provided a full explanation of how to access the data, including relevant URLs and DOIs, in the revised cover letter. We have also provided this in the Data Availability section of the online submission system.**

Reviewers' comments:

Reviewer 1

Reviewer #1: Thank you for the opportunity to review the article “The experiences of UK-Chinese individuals during the COVID-19 pandemic: A qualitative study”. The paper aims to investigate the lived experiences of Chinese individuals living in the UK during the COVID-19 pandemic. I have enjoyed reading some of the empirical findings but think substantial improvements are needed.

**Author response: Thank you for taking the time to review our manuscript. We are happy to hear you enjoyed reading some of the findings. We appreciate your suggestions to improve our manuscript. We believe that our amendments detailed below address the concerns you have raised and our manuscript has been strengthened as a result.**

1. Research aim/design: I find the research aim “to investigate the lived experiences” a bit too broad and vague. There are too many aspects of lived experiences even when specified to COVID. Reflecting this, the interview questions (S1) are also rather general and unfocused. What was the underlying rationale of asking these questions? Why are the ‘family and overseas’ and ‘the future’ relevant? What did the authors want to find in the first place?

**Authors’ response: Thank you for these comments. We agree that the statement in our abstract/introduction that we aimed to “investigate the lived experiences” is too broad and vague. Our primary aim was to explore the impact of the pandemic on the UK-Chinese community, specifically to find out how perceived cultural and ethnic identity impacted experiences of the pandemic, and how these experiences compared to those of family or friends in China/East Asia.**

**We were also interested to explore the extent to which behaviours and practices were influenced by practices in Mainland China. As such, the ‘Family and overseas’ section was relevant to explore how UK-Chinese individuals received information from China/East Asia, their perceptions of the different responses (both societally and governmentally) in the context of the pandemic and the extent to which this shaped their behaviours in the UK. Family and relatives experiences in China/East Asia acted as comparators to the experiences of the study participants. Likewise, the ‘Future’ topic investigated participants’ perspectives on the lasting impact of the pandemic. We have clarified and expanded on the study aims in the abstract:**

***“Infectious disease outbreaks have historically been associated with stigmatisation towards minority groups, specifically those associated with the geographical region that the disease was first identified. We aimed to investigate how the emerging COVID-19 pandemic was experienced by UK-resident individuals of Chinese ethnicity: how their perceived cultural and ethnic identity influenced their experiences, and how early insights into the pandemic in China influenced attitudes and behaviours.”***

**We have also restated the aims of our study in the introduction to now read:**

***“We aimed to investigate i) how perceived cultural and ethnic identity impacted the experiences of UK-Chinese individuals during the pandemic, ii) how behaviours and practices of UK-Chinese individuals were influenced by insight into the pandemic responses in China/East Asia, and iii) the perspectives of UK-Chinese individuals on the long-term impacts of the pandemic on the UK-Chinese community.”***

**We agree the questions presented in the topic guide are general. The guide was designed to list the broad topics to be covered during the interview, and to guide the interviewer in their questioning. The questions listed were not asked verbatim to participants. A long and detailed guide was not considered practical to use in these interviews. The guide had to allow flexibility in specific question construction, order, and timing. We have updated the methods section to state this which now reads:**

***“A flexible topic guide was developed by the study team (S1 File). This served as a broad guide of topics to discuss: the questions listed were not asked verbatim to participants and evolved as interviews progressed.”***

2. Significance/study contribution: Although little research has been conducted to understand “the experiences of UK-Chinese individuals in the UK early in the pandemic”, there is a large literature on anti-Chinese/Asian sentiment and racism in general. And earlier studies have also investigated this issue during previous pandemics such as SARS. How does this study add any new insight to these two strands of research?

**Authors’ response: Thank you for this comment. We have edited our manuscript to better highlight the new insights provided by our research, and place our work in the context of the existing literature on anti-Chinese/Asian sentiment. Since the submission of our manuscript, some further relevant papers have been published which we have also discussed and referenced in our manuscript.**

**The new insights provided by our study are summarised:**

**a) the experiences of people of Chinese ethnicity in the UK/Europe in the context of the COVID-19 pandemic have been poorly described, with most of this research stemming from the USA/American continent. Although there may be commonalities, the experiences are context and individual specific, and so the experiences of US Asians will have limited transferability to a UK-Chinese population**

**b) we provide an in-depth description of participants’ experiences in their own words. There is very little rigorous qualitative research that has explored the experiences of people of Chinese/Asian heritage living outside of Asia during the COVID-19 pandemic. The vast majority of existing research, most of which stems from the USA as mentioned above, is limited to quantitative surveys. Much of this existing literature we have now referenced in our manuscript. However the information derived from surveys is considerably less rich or in-depth than that collected through qualitative research. We have identified only 3 relevant qualitative studies, although in notably different contexts, which we have also referenced in our manuscript, the most relevant of which is a study of experiences of healthcare workers of Asian heritage in North America [Reference doi:10.9778/cmajo.20210090].**

**c) our research was undertaken in a country essentially unaffected by SARS, but severely impacted by COVID-19. There were only 4 UK cases of SARS, so while the experience in other countries affected significantly by both infections may have been similar, the UK did not have this experience. The largest outbreak outside Asia was in Canada. The most relevant study is cited as reference 3 in our manuscript [Reference doi:10.3201/eid1002.030750], however this study incorporated a range of measures to gauge anti-Asian sentiment in the USA including reviewing newspapers and internet sites, group discussions and analysing phone calls to a Centre for Disease Control telephone service. What interviews that took place were described as “informal”, and there was no formal analysis of these. As such, we feel that the strand of research into anti-Asian sentiment in the context of SARS is limited, and lacks qualitative insight.**

**d) the timing of our research study provided contemporaneous insight into the experiences of this group as the pandemic emerged, and compared the experiences of UK-Chinese individuals with those of family members and contacts in China/East Asia, and compared governmental responses, in real time.**

**f) with regards to what this study adds to existing research around anti-Chinese/Asian sentiment generally, a key insight is that participants in this study described racism and stigmatisation related to infection control behaviours in response to the pandemic (e.g. face masks), as well as physical features/perceived ethnicity. Related to this, it offers new and we believe interesting insight into how decisions regarding adopting of these practices were made – and how this was balanced against the risk of stigmatisation. We have also now included some discussion of our findings in the historical context of orientalism and sinophobia, and the role of the media in perpetuating this.**

**We have made substantial changes to our discussion, and expanded our conclusions, to address the points you have raised and hope that the new insights our study provides are now more clear.**

*3. Sampling: The authors have relied on a very small sample which overwhelmingly consisted of people who were born in Hong Kong. To what extent are the findings from this sample generalisable to the UK-Chinese population? I am very suspicious about whether people who were born in Hong Kong and people who were born in Mainland China share the same feelings and attitudes especially when it comes to their family and relatives’ experiences in their home country (Hong Kong vs Mainland China). The inclusion of only 4 individuals who were born in Mainland China seems very problematic.*

**Authors’ response: Thank you for these comments.**

**We aimed to achieve a diverse sample, rather than a population representative sample which is associated with quantitative research. Qualitative studies seek to provide in-depth and diverse insights into a phenomenon and do not attempt to generalise the results to a population of interest [Reference https://pubmed.ncbi.nlm.nih.gov/10891968/]. Most qualitative studies are necessarily small scale to provide depth and contextualised detailed information, and thus the criterion of generalisability (external validity) cannot be applied. Instead, transferability is the extent to which the concepts and theories are relevant to other settings. Researchers can compare their results with studies conducted in different healthcare contexts, regions, or populations; position their findings with other theoretical frameworks; and describe the study setting and participant characteristics in detail so readers can judge the transferability of the findings to their own context [Reference https://journals.lww.com/transplantjournal/Fulltext/2016/04000/Qualitative_Research_in_Transplantation__Ensuring.9.aspx].**

**In our study sample, we achieved diversity with respect to sex, age, educational level, place of birth and year of migration to the UK. We have provided participant details to allow readers to judge the transferability of the findings to other individuals in other contexts. We recognise that findings may have limited transferability to other Chinese populations, and this was not an aim. We have added a statement to the limitations section stating that findings from this study may not transfer to a population of Chinese individuals from other countries.**

**The UK-Chinese population is a heterogenous group comprising individuals who self-identify as being of Chinese ethnicity, and includes individuals who are of Chinese heritage born in the UK, and individuals of Chinese heritage born outside the UK who have migrated to the UK. The most recent UK census states that only 23.7% of people of Chinese ethnicity were born in the UK [Reference https://www.ethnicity-facts-figures.service.gov.uk/summaries/chinese-ethnic-group] which is just above the proportion of UK-born participants in our study. Hong Kong was until 1997 a UK territory, and the majority of emigrees of Chinese ethnicity to the UK are from Hong Kong. The inclusion of fewer individuals born in Mainland China reflects the UK-Chinese population as a whole.**

**We acknowledge that governmental responses in Mainland China and Hong Kong need to be distinguished, and your concern that individuals born in Mainland China and Hong Kong may not share similar feelings or attitudes with regards to their families’ experiences. The measures initially imposed in Hong Kong were less restrictive than in mainland China, but there were still strict quarantine and isolation requirements earlier than when introduced in the UK. However, there is primary research from Hong Kong that describes how individuals there adopted more stringent measures – more in line with the control measures in Mainland China - out of choice. For example, mask-wearing in Hong Kong was near universal in January 2020 despite it not becoming mandatory until July 2020 [Reference doi:10.12809/hkmj209015; Reference doi:10.1016/s2468-2667(20)30090-6]. We have edited our manuscript to state to the reader that responses in China and Hong Kong differed. This section reads:**

***“The measures taken in mainland China to control the pandemic, while effective, were described as ‘draconian’ in a qualitative study of rural villagers in Hunan, China [35]. As a semi-autonomous region within China, the initial response in Hong Kong diverged, and was less restrictive, with face mask use not becoming mandatory until July 2020 [36]. However, a cross-sectional survey of behaviours in Hong Kong in the very early stages of the pandemic described widespread voluntary adoption of stringent infection control measures, such as isolation and face mask use [13].***

4. Findings/analysis: Overall, I find the findings scattered, which is linked to my previous comment on the lack of focus in research aim/design. Some of the findings are interesting (e.g., the participants did not explicitly show preference between the British and Chinese governments; an implicit faith in government) but very limited in-depth insight has been generated.

**Authors’ response: Thank you. On review of our manuscript, and in light of your previous comments, we feel that the findings seemed scattered in large part because our aims were too broadly stated and unfocussed. By stating our aims more clearly, as above, we hope this has been partly addressed. We have also made our analysis more focussed by restating and condensing some of the themes, and amending the discussion to tie in with our stated aims more closely.**

**We are happy to hear that you found some of the findings interesting, and believe that the amendments we have made in light of your comments have highlighted some of our other findings. We have more clearly stated the new insights generated, and included greater discussion of how our research adds to existing literature around anti-Asian/anti-Chinese racism generally, and in the context of infectious disease pandemics, including SARS and COVID-19. Importantly, we have highlighted the importance of describing the negative experiences of this community, and factors that may have exacerbated this, such that lessons can be learned in future infectious disease outbreaks.**

**Thank you again for taking the time to review our manuscript, and we hope that the improvements we have made in response to your comments are satisfactory.**
