# Supplementary material for: The experiences of UK-Chinese individuals during the COVID-19 pandemic: A qualitative interview study
Source: PLoS One. 2023 Jan 17;18(1):e0280341. doi: 10.1371/journal.pone.0280341 (PMC9844865; doi:10.1371/journal.pone.0280341)
Supplement: Attachment — Submitted filename: Dear Editor.docx [file pone.0280341.s005.docx]

Dear Editor

I have read the article entitled ***The experiences of UK-Chinese individuals during the COVID-19 pandemic: a qualitative interview study*** and I find that the subject, although it is pertinent, the development of the article does not have sufficient quality. It is not clear why such a limited population group is interviewed. When using the qualitative method, it is necessary to have data saturation. Likewise, the categories of analysis are not supported in a strong literature review, necessary to carry out the discussion and conclusions.

The important thing about the literature review is that it is the basis for dialogue with the findings. However, in this section the authors limit themselves to a description without greater depth of the responses of the participants and leave aside the rigorous analysis.

On the other hand, in the bibliography the authors refer to 41 documents, however in the document there are no more than 20.

In conclusion, I consider that the article does not have the requirements to be published in such a prestigious journal.

Sincerely,

Lida Villa
