# Supplementary material for: The experiences of UK-Chinese individuals during the COVID-19 pandemic: A qualitative interview study
Source: PLoS One. 2023 Jan 17;18(1):e0280341. doi: 10.1371/journal.pone.0280341 (PMC9844865; doi:10.1371/journal.pone.0280341)
Supplement: Attachment — Submitted filename: Response to reviewers.docx [file pone.0280341.s006.docx]

**Editor's comment to the author:**

**The reviews outcome from the two reviewers of your paper are conflicting. One of the reviewers--a new reviewer for your paper--is very negative, but the comments he made is a bit general.**

**Although those negative comments are not very specific, I believe that you should address seriously the issues raised by the reviewer, if you want to proceed this review process.**

**I also reviewed the paper by myself, and I still think it is an interesting work, and bringing new understanding for this topic. In your manuscript, you discussed that the UK-Chinese individuals experienced and perceived widespread xenophobia, in the context of media representations that ascribed blame and exacerbated stigmatisation. This in turn marked UK-Chinese individuals as targets for abuse. Participants described media representations of China as apportioning blame and contributing to  xenophobia.  For the media representation of China in western society and how this has contributed to forms of violence, I suggest to include the following paper:**

**‘Can Debunked Conspiracy Theories Change Radicalized Views? Evidence from Racial Prejudice and Anti-China Sentiment Amid the COVID-19 Pandemic’, Journal of Chinese Political Science, DOI: 10.1007/s11366-022-09832-0**

**In your discussion, you mentioned that some of your findings are supported by a study of villagers in Hunan and a survey in Hubei province. Although these values cannot be directly ascribed to UK-Chinese people, you found a consistent theme of implicit faith in government among your interviewees.  I think it will be helpful to recognize and consider the differences of implicit faith between urban and rural societies in China, and between different rural communities in China. For this, it may be helpful to read the following paper:**

**‘Fragmented Restriction, Fractured Resonances: Grassroots Responses to Covid-19’, Critical Asian Studies. 52(4): 494-511**

Authors response: Thank you for these suggestions. We have reviewed these papers and have referenced them in our discussion.

**Reviewer comments**

**Reviewer 1**

Authors’ response: We’re pleased that Reviewer 1 feels our responses to their comments are acceptable, and recommended that the manuscript be accepted.

**Reviewer 2**

**I have read the article entitled The experiences of UK-Chinese individuals during the COVID-19 pandemic: a qualitative interview study and I find that the subject, although it is pertinent, the development of the article does not have sufficient quality. It is not clear why such a limited population group is interviewed.**

Authors’ response:

Our sample was diverse with respect to age, gender, educational attainment and place of birth/emigration to the UK. Sampling was undertaken during the COVID-19 pandemic and lockdowns in the UK. We acknowledge the possible limitations of convenience and snowball sampling in our discussion section: “Our sampling strategy may have limited the diversity of participants of the study, although participants were diverse in terms of age, educational background and gender.”

**When using the qualitative method, it is necessary to have data saturation.**

Authors’ response: As indicated in our methods we used Braun and Clarke’s method of thematic analysis. Braun and Clarke state that the concept of data saturation is not consistent with the values and assumptions of reflexive thematic analysis: V Braun and V Clarke. To saturate or not to saturate? Questioning data saturation as a useful concept for thematic analysis and sample-size rationales. Qualitative Research in Sport, Exercise and Health 2021;13(2): 201-16. <https://www.tandfonline.com/doi/full/10.1080/2159676X.2019.1704846>.

Braun and Clarke suggest that other concepts such as ‘information power’ offer a more useful way of assessing data samples. Malterud, Siersma, and Guassora (2016) conclude that the more relevant information a sample holds, the fewer participants are needed. We have added the following sentence to our manuscript: ‘We assessed that the sample delivered sufficient information power, as the information provided in the interviews was rich, highly relevant and included contemporaneous accounts of current lived experiences.’

**Likewise, the categories of analysis are not supported in a strong literature review, necessary to carry out the discussion and conclusions.** **The important thing about the literature review is that it is the basis for dialogue with the findings. However, in this section the authors limit themselves to a description without greater depth of the responses of the participants and leave aside the rigorous analysis.**

Authors’ response: In response to suggestions from the Editor, we have discussed two other papers from the literature and added these to the reference list. We feel our discussion is rich and considers our findings in the context of the limited existing literature. As we note in the manuscript, to our knowledge, this is the first qualitative study to rigorously explore the experiences of people of Chinese ethnicity in the UK with regards to the COVID-19 pandemic. Indeed, there is limited qualitative research exploring the experiences of individuals of Chinese or Asian ethnicity in the Western world during COVID-19 or previous infectious disease outbreaks, such as SARS in 2003. There is therefore limited directly comparable literature with which to compare findings.

**On the other hand, in the bibliography the authors refer to 41 documents, however in the document there are no more than 20.**

Authors’ response: We can only assume that this comment was made in error. All 41 references in the reference list were referenced in the text.
